# Comprehension strategy instruction for Hispanic children with mathematical learning difficulties

**DOI:** 10.3389/fpsyg.2025.1645323

**Published:** 2025-11-04

**Authors:** Michael J. Orosco, Shahlo Mamedova, Naheed A. Abdulrahim

**Affiliations:** ^1^Educational Psychology, University of Kansas, Lawrence, KS, United States; ^2^Teacher Education, University of Nebraska Kearney, Kearney, NE, United States

**Keywords:** Hispanic students, language minority students, comprehension strategy instruction, working memory, reading comprehension, problem-solving, educational neuroscience, mathematical learning difficulties

## Abstract

Hispanic children face significant problem-solving challenges due to the simultaneous linguistic and cognitive demands of mathematics. This study examined the effectiveness of comprehension strategy instruction (CSI) for Hispanic children and investigated working memory as a potential moderator of intervention effectiveness. Participants included 43 third-grade Hispanic children with mathematical learning difficulties and 32 control peers from an urban public school system in the southwestern United States. Using hierarchical linear modeling, results demonstrated significant effects of CSI on mathematical performance, with medium effect sizes across multiple assessments. An interaction model revealed that both CSI and working memory were significant predictors of mathematical achievement, with a marginally significant two-way interaction implying that students’ cognitive profiles moderated the impact of the intervention. Findings highlight the importance of integrating CSI into mathematics instruction for Hispanic students to support both linguistic and cognitive development.

## Introduction

Hispanic children constitute a significant demographic in U. S. public schools, with approximately 13.6 million enrolled as of Fall 2021, representing 28% of the total student population (National Center for Education Statistics, 2024a). Although most receive instruction predominantly in English, only about 10% participate in bilingual education programs (Takanishi and Le Menestrel, 2017). These students function as language minority learners (LMLs), also known as English Learners (ELs). They represent a diverse language development spectrum. This spectrum ranges from limited English proficiency to fully bilingual status, including English-dominant children who maintain some Spanish proficiency (August and Shanahan, 2006). Many Hispanic children come from low-income households, which is known as a risk factor for compromised academic performance. National data reveal persistent achievement gaps in mathematical performance. In 2022, Hispanic fourth-grade children scored 25 points lower in mathematics than their non-EL peers. This gap widens significantly to 41 points by eighth grade (National Center for Education Statistics, 2024b). These discrepancies underscore the importance for evidence-based mathematics interventions specifically designed for multilingual learners.

A critical challenge for Hispanic children is solving mathematical word problems, which simultaneously require mathematical reasoning, reading comprehension, and language skills. These multiple demands place a high cognitive load on working memory (WM), making problem-solving particularly challenging for students acquiring English. Developing problem-solving skills among students at the elementary level presents significant challenges, particularly when solving complex mathematical word problems in their second language without native language support. This challenge is magnified by demographic trends in the United States, where ELs constitute a rapidly growing segment of the public-school population. Research consistently documents achievement gaps in mathematics between ELs and their monolingual peers (NASEM, 2018).

Addressing these mathematical disparities requires instructional strategies grounded in a cognition framework that integrates comprehension strategy instruction (i.e., CSI, reading development, language skills, and cognitive supports) to enhance problem-solving efficiency (Orosco and Reed, 2023). This framework recognizes the interconnected roles of working memory, language proficiency, and reading comprehension in mathematical reasoning. Effective interventions may include integrating mathematical practices with reading skills and oral language development, implementing visual supports, and adapting mathematical tasks to be more accessible for students acquiring the language of instruction (e.g., Orosco and Reed, 2023, 2024; Swanson et al., 2025). These targeted approaches, that address both linguistic and mathematical needs, are essential in addressing student academic achievement among Hispanic children and subsequent long-term educational success.

### Working memory and reading comprehension: interrelated predictors of mathematical performance

Within this framework, working memory (WM) capacity and reading comprehension function as interrelated predictors that significantly impact mathematical achievement. Working memory, defined as a domain-general cognitive function essential for temporarily storing and manipulating information, plays a critical role in solving multi-step word problems (Baddeley, 2012). Research has demonstrated that WM capacity significantly impacts mathematical problem-solving performance, particularly in linguistically complex scenarios where language skills are required to decode, comprehend, and formulate responses to mathematical problems (Swanson et al., 2010; Van der Ven et al., 2013). Concurrently, reading comprehension provides the essential framework for interpreting and translating mathematical problem text (Fuchs et al., 2018; Träff et al., 2025; Vukovic and Lesaux, 2013).

Hispanic children engaging with word problems face unique challenges when working in their non-dominant language. Under these conditions, the relationship between working memory and reading comprehension becomes particularly consequential. As working memory simultaneously addresses linguistic processing demands and mathematical operations, this can result in a cognitive bottleneck and impede problem-solving performance (Swanson et al., 2015). When reading comprehension skills are underdeveloped, working memory resources compensate by emphasizing basic text decoding rather than mathematical reasoning (Fuchs et al., 2018; Swanson et al., 2015). This compensation further constrains problem-solving capacity (Bernardo and Calleja, 2005). This synergistic relationship between working memory and reading comprehension suggests that interventions targeting both cognitive efficiency and linguistic comprehension may yield a multitude of benefits for mathematical achievement among Hispanic children (Geary et al., 2012; Peng et al., 2020). Students with higher working memory capacity can more effectively utilize reading comprehension strategies, whereas enhanced reading comprehension reduces the cognitive load imposed on working memory during problem-solving tasks.

Consider a sample problem: “A baker starts with 30 cookies, sells 12 in the morning and eight in the afternoon, bakes 15 more in the evening, and gives five to a neighbor—how many are left?.” This problem demonstrates multiple cognitive demands: children must not only read for understanding but simultaneously hold and manipulate multiple numerical components while filtering irrelevant details. The problem requires students to maintain temporal sequence (morning, afternoon, evening), track multiple operations (subtraction and addition), and distinguish relevant from irrelevant information—all while processing the linguistic components of the word problem in their second language. Students with higher WM capacity may be better equipped to utilize instructional supports and process complex problem structures effectively. These learners can typically maintain active representations of both the problem’s linguistic and mathematical components while implementing solution strategies (Swanson et al., 2018). Their enhanced cognitive resources allow them to simultaneously process the semantic meaning of the text, maintain running calculations, and implement problem-solving heuristics more efficiently.

Conversely, those with lower WM capacity may experience cognitive overload, potentially leading to difficulties in problem-solving performance (Paas and Van Merrienboer, 2020). This cognitive strain can manifest in several ways: difficulty maintaining intermediate results, confusion about operation sequence, and challenges in distinguishing relevant from irrelevant information (Meyer et al., 2010). The combined demands of cognition processing and mathematical comprehension can overwhelm the limited WM resources, particularly when problems involve multiple steps or complex linguistic structures (Raghubar et al., 2010). Research suggests that the relationship between WM capacity and mathematical problem-solving is particularly pronounced for Hispanic children, who must dedicate additional cognitive resources to language processing. This cognitive burden may be especially evident in word problems that contain multiple transformation steps. These steps require sequential processing, irrelevant information that must be filtered out, complex linguistic structures or academic vocabulary, temporal sequences that must be maintained throughout problem-solving, and mixed operations requiring careful attention to mathematical signs and procedures (Vukovic and Lesaux, 2013).

Given the complex interplay between linguistic processing, working memory capacity, and mathematical reasoning documented in the literature, targeted interventions must address these interconnected cognitive demands. The current study investigates whether comprehension strategy instruction can effectively support Hispanic children with mathematical learning difficulties by addressing both linguistic and cognitive barriers simultaneously. This investigation addresses the following research questions:

1. Does integrating comprehension strategy instruction improve word-problem accuracy and overall mathematical achievement among Hispanic children?
2. Does working memory (WM) capacity and reading comprehension moderate the effectiveness of comprehension strategy instruction on word-problem performance among children?

### Hypothesis one (H1)

We hypothesize that Hispanic children who receive teaching that integrates comprehension strategy instruction will demonstrate significantly higher word-problem accuracy and overall achievement compared to children who receive standard mathematical instruction without explicit comprehension supports. The rationale for this hypothesis is built upon several key areas. First, research on comprehension-based interventions consistently demonstrates their effectiveness (e.g., Orosco and Reed, 2023, 2024; Orosco and Abdulrahim, 2018; Kong and Orosco, 2016; Orosco et al., 2013). These interventions, which include explicit vocabulary instruction, reading comprehension scaffolds, and structured problem-solving techniques, improve students’ ability to decode mathematical language, understand problem structures, and engage in effective problem-solving (Fuchs et al., 2019). Supporting evidence demonstrates that combining reading and mathematical comprehension strategies, with explicit focus on decoding, phonological awareness, and numeracy, significantly enhances children’s understanding and application of mathematical concepts (Orosco and Reed, 2023, 2024; Orosco and Abdulrahim, 2018; Kong and Orosco, 2016; Orosco et al., 2013). Second, integrating comprehension strategies directly addresses the linguistic barriers that often hinder children’s ability to read and understand mathematical problems (NASEM, 2018). Research emphasizes the importance of focusing on relevant language within word problems and providing collaborative support to improve children’s solution accuracy. Finally, direct cognitive instruction, such as using overt cues and structured problem-solving strategies, supports cognitive load management and working memory efficiency, leading to enhanced mathematical problem-solving, particularly for at-risk learners (Fuchs et al., 2020).

### Hypothesis two (H2)

We hypothesize that the effectiveness of comprehension strategy instruction in enhancing word-problem performance among children will depend on students’ WM capacity. Specifically, children with higher WM capacity are expected to benefit more from integrated instruction. Conversely, children with lower WM capacity may require additional instructional scaffolds, such as chunking information, providing multimodal supports, or using worked examples, to maximize their learning potential. The rationale for this hypothesis centers on the well-established link between WM and academic performance. Research consistently demonstrates that WM capacity is a significant predictor of mathematical achievement, especially in word problem-solving (Geary, 2011). This aligns with findings highlighting WM’s contribution to reading comprehension, particularly in processing and manipulating the information needed for complex tasks. Furthermore, WM is closely linked to language skills. Strong WM capacity allows students to process and integrate linguistic and mathematical information more efficiently (Peng et al., 2020). Studies suggest that WM capacity influences the strategies students use to learn, with those possessing higher WM capacity employing a wider range of strategies, including top-down approaches that facilitate global comprehension (e.g., Alloway and Alloway, 2010; Foster et al., 2017; Peng et al., 2018; Swanson, 2003). WM constraints can negatively impact students’ ability to translate mathematical word problems into appropriate representations, leading to difficulties in procedural execution and accuracy (Van de Weijer-Bergsma et al., 2015). Therefore, differentiated instruction becomes crucial for maximizing learning outcomes. Instructional strategies should be systematically tailored to accommodate students’ working memory capacity through structured routines, visual organizers, and multimodal instruction that can mitigate WM overload (Paas and Sweller, 2014).

### Conceptual framework

This study applies an integrated framework from comprehension strategy instruction (CSI) and cognitive psychology research to examine how Hispanic children process mathematical word problems. By investigating both language acquisition and cognitive processing demands, the research addresses the complexities children encounter when solving mathematical problems in their non-native language. This framework underscores the connection between conceptual understanding, procedural fluency, and problem-solving efficiency (National Research Council, 2001).

The CSI intervention directly addressed Cognitive Load Theory principles by reducing extraneous cognitive load through explicit vocabulary instruction, optimizing intrinsic load via structured information analysis, and enhancing germane load through collaborative practice that promoted schema construction. For students with limited working memory capacity, these modifications prevented cognitive overload that typically impedes mathematical problem-solving performance. Cognitive Load Theory (Sweller, 1988; Sweller et al., 2019) provides an essential foundation for understanding how working memory (WM) constraints affect children’s problem-solving abilities. Word problems require simultaneous linguistic decoding, retention of numerical information, and sequential problem execution, which can overwhelm children’s WM capacity (Ayres and Sweller, 2022). To optimize WM efficiency, instructional strategies should minimize extraneous cognitive load, leverage multimodal supports, and use scaffolded repetition with increasing complexity to reinforce problem-solving fluency.

Working memory and reading comprehension represent two critical, interconnected cognitive processes that significantly impact mathematical achievement among Hispanic children. Research has consistently demonstrated that these two factors not only individually predict mathematical performance but function synergistically within the problem-solving process (Peng and Fuchs, 2016). Working memory serves as the cognitive foundation that enables the complex processes involved in reading comprehension, while reading comprehension skills provide the framework for interpreting and translating mathematical problem text (Carretti et al., 2009). For Hispanic children engaging with word problems in their non-dominant language, this relationship becomes particularly consequential. Working memory must simultaneously manage linguistic processing demands and mathematical operations, creating a potential cognitive bottleneck that can impede performance (Swanson et al., 2015). When reading comprehension skills are underdeveloped, working memory resources become disproportionately allocated to basic text decoding rather than mathematical reasoning, further constraining problem-solving capacity (Bernardo and Calleja, 2005). Conversely, stronger reading comprehension skills can compensate for working memory limitations by facilitating more efficient text processing, allowing cognitive resources to be redirected toward mathematical operations (Fuchs et al., 2018).

The bidirectional relationship between these factors suggests that interventions targeting both working memory efficiency and reading comprehension strategies may yield multiplicative benefits for mathematical achievement among Hispanic children (Geary et al., 2012; Peng et al., 2020). Students with higher working memory capacity can more effectively deploy reading comprehension strategies, while enhanced reading comprehension reduces the cognitive load imposed on working memory during problem-solving tasks. This interdependence forms the theoretical foundation for our integrated instructional approach, which explicitly addresses both cognitive and linguistic dimensions of mathematical problem-solving. Research suggests that integrating explicit vocabulary instruction, comprehension scaffolds, and structured problem-solving techniques can enhance children’s ability to decode mathematical language and interpret problem structures effectively (Lesaux et al., 2010). Given the role of WM in problem-solving, instructional scaffolds such as chunking, multimodal supports, and worked examples can help mitigate cognitive overload, particularly for children with lower WM capacity (Swanson and Gerber, 2013; Sweller et al., 2019).

Synthesizing principles from cognitive and linguistic frameworks, this study proposes a four-component integrated instructional model for children: (a) language supports (explicit vocabulary instruction and comprehension scaffolds), (b) cognitive scaffolds (structured routines and chunking techniques), (c) problem-solving strategies (step-by-step guided instruction and worked examples), and (d) equitable practices (differentiated instruction tailored to children’s linguistic and cognitive profiles). Research has shown that direct cognitive instruction enhances mathematical problem-solving among at-risk learners by supporting cognitive load management and WM efficiency (Barbieri and Rodrigues, 2025; Swanson et al., 2015). Similarly, Peng et al. (2020) highlight the bidirectional relationship between WM and language skills, emphasizing the importance of integrating cognitive supports in literacy-based mathematical instruction for children. By addressing the interplay of language development and cognitive processing, this study provides a robust framework for designing evidence-based instructional strategies for children. This approach aligns with research demonstrating that structured routines, visual organizers, and multimodal instruction can enhance problem-solving efficiency by reducing WM overload (Paas and Sweller, 2014). As children navigate the dual demands of mathematical reasoning and linguistic comprehension, instructional interventions grounded in cognitive and linguistic supports are critical for closing achievement gaps and improving mathematical outcomes.

## Method

The research protocol involving human participants was reviewed and approved by the first author’s university’s institutional review board. Approval documentation is maintained by the institution during the study period, and specific dates are available upon request from the institution. Full approval was obtained prior to participant recruitment, with all procedures complying with institutional and federal ethical requirements at the time of data collection. Additional permissions were obtained from both the school district administration and the students’ parents prior to study commencement.

### Participants

Study participants included 75 Hispanic English Learners (43 with mathematical learning difficulties in the treatment group and 32 in control group; males = 38, females = 37; *M* = 7.94 years, *SD* = 0.42). All participants were drawn from mainstream English-only elementary classrooms within an urban public school system in the southwestern United States. According to school records, every participant was eligible for the free or reduced-price meal program. Early Intermediate to Advanced, participants demonstrated a range of English proficiency levels as measured by their district English language development assessment. Across these developmental stages, they varied in accuracy of English usage, comprehension of concrete information, and capacity to engage with abstract concepts during extended academic discussions. Instruction consisted exclusively of English-only teaching across all content areas, including mathematics, with additional English as a Second Language (ESL) support provided throughout the school day.

### Operational definition of serious math learning difficulties

For this investigation, we established a definition of serious mathematical learning difficulties (MLD) through a sequential two-phase screening protocol, as most participants lacked formal diagnoses of specific learning disabilities in mathematical problem solving. The preliminary screening involved teacher identification based on three key criteria: students receiving mathematical instruction in English, performance below district benchmarks on standardized reading and math assessments and observed difficulties with problem-solving tasks despite general education instruction. For the secondary screening phase, the researchers administered a self-developed mathematical word problem assessment that mirrored the participants’ state’s standardized mathematics test (California Department of Education, 2013). These Grade 3-level problems aligned with standards from the National Council of Teachers of Mathematics (2014) and required students to interpret mathematical language, identify relevant information, and apply mathematical operations across domains including number operations, measurement, geometry, and data analysis. Participants scoring below the 25th percentile on this assessment met inclusion criteria for mathematical learning difficulties classification (Geary et al., 2012; Lambert and Spinath, 2018; Mazzocco et al., 2013). Furthermore, participants were required to demonstrate sufficient working memory capacity, as measured by the S-Cognitive Processing Test (Swanson, 1995), and foundational comprehension strategy skills, evaluated through informal reading comprehension tasks. These criteria functioned as control variables to ensure that all participants possessed the cognitive prerequisites necessary for valid assessment of mathematical problem-solving performance. This protocol identified 43 Hispanic children experiencing mathematical learning difficulties who were subsequently compared with 32 peers without such difficulties. To control for potential instructional variables, we ensured both intervention and control groups were randomly assigned across the mainstream classrooms participating in the study. The targeted intervention applied comprehension strategy instruction (CSI) to address the unique challenges faced by Hispanic children navigating both linguistic and cognitive demands in mathematical problem-solving contexts.

#### General education instruction

Qualitative classroom observations were conducted by a two-person research team with advanced degrees specializing in ESL and bilingual mathematics pedagogy to document instructional methodologies (Gámez and Lesaux, 2015). The primary researcher, who possesses a doctoral degree in bilingual special education, collaborated with a co-observer to document comprehensive field notes encompassing instructional activities, classroom environment configurations, and teacher-student discourse patterns, along with their analytical reflections. Methodological rigor was strengthened through systematic debriefing protocols and participant validation procedures (Kibler et al., 2015). The observational data collection spanned a three-month period, comprising multiple instructional sessions of 50 min each. Educational content was delivered primarily by general education teachers implementing the district-adopted standards-aligned curriculum materials. Control group teachers were not informed of specific intervention content to prevent contamination. Classroom observations confirmed that no systematic comprehension supports beyond standard curriculum materials were provided. Instructional time was equivalent across conditions (50 min daily), with content aligned to identical district standards and pacing guides. Daily mathematical problem-solving instruction followed a bifurcated structure: an initial 25-min teacher-directed instructional segment followed by a 25-min application phase focused on independent student practice. Although worked examples were incorporated into the instructional approach, the absence of clearly articulated proficiency criteria frequently resulted in comprehension challenges among students—particularly those in the intervention group—when encountering unfamiliar terminology and linguistically complex word problems.

#### Supplemental intervention

A supplementary instructional program was delivered using a pullout model that complemented the core mathematics curriculum. The 43 students identified with mathematical learning difficulties were allocated through randomized assignment into instructional clusters of three to five participants following their identification through educator recommendations and performance metrics on standardized assessments (including both word-problem solving instruments and reading comprehension measures). The randomization procedure, stratified according to participants’ mathematical and literacy performance rankings, was designed to prevent concentration of students with the most significant challenges within any single group. The supplementary curriculum content aligned with problem typologies addressed in the standard instructional program (e.g., combination, separation, part-part-whole relationships, and comparison scenarios) and was presented through structured materials comprising 20 instructional units, each containing five problem scenarios. Implementation was conducted by graduate-level instructors with specialized training in elementary education methodology, ESL instruction, and special education practices. The program was administered on a semi-weekly schedule throughout a 10-week intervention period. Each 30-min instructional session followed a balanced structure, allocating approximately 15 min to explicit instructional delivery followed by 15 min of supported independent application. Students were first taught and initially applied strategies using basic word problems. They advanced to more complex problems requiring continued strategy instruction only after demonstrating complete understanding and accurate application of the strategies.

#### Intervention Fidelity

All tutors underwent an intensive four-hour professional development workshop on implementing the 20 structured lesson protocols. Instructors engaged in peer practice sessions and received direct supervision until demonstrating 90% proficiency on a standardized assessment tool. During the intervention, treatment fidelity was evaluated by observers (e.g., first author or project director) who independently completed evaluation forms covering all lesson segments. Observations occurred for six randomly distributed sessions per tutor, with interrater agreements exceeding 90%. Tutors followed each implementation step with scores above 95% across all sequences, conditions, and sessions. Additional coaching was provided to any instructor falling below the performance benchmark. Student advancement required demonstrated proficiency in strategy application during guided practice before transitioning to autonomous activities.

##### Intervention procedure

The intervention explicitly addressed working memory constraints through chunking information into manageable segments during Phase 1, providing visual–spatial supports during Phase 2 to reduce cognitive load, and implementing collaborative structures in Phase 3 to distribute cognitive demands. Reading comprehension was systematically targeted through three primary instructional components embedded throughout all four phases: explicit vocabulary instruction, text structure analysis, and comprehension monitoring strategies. Text structure analysis specifically involved identifying problem types, recognizing linguistic signals that indicate mathematical operations, and understanding temporal and causal relationships within problem narratives. The intervention followed a structured four-phase protocol.

##### Phase 1: problem understanding

Instructors explicitly modeled comprehension strategies for interpreting word problems, helping students make connections, ask questions, visualize, synthesize, and monitor their thinking. Instructors read problems aloud while demonstrating how to identify the question, develop a mental model, restate the question in students’ own words, and record complete problem statements in workbooks.

##### Phase 2: information analysis

Students were taught to analyze problem components through strategic text marking, distinguishing between relevant and irrelevant information, activating prior knowledge, and creating appropriate visual representations of mathematical relationships. Students learned to conceptualize word problems as belonging to specific types and represent each type with a diagram or equation.

##### Phase 3: collaborative practice

Structured peer collaboration was implemented where students practiced applying strategies with partners while receiving instructor guidance. Students verbalized their problem-solving processes while instructors assessed understanding through strategic questioning and provided immediate corrective feedback. This collaborative support significantly improved students’ solution accuracy in mathematical word problem-solving contexts.

##### Phase 4: independent application

Students independently solved similar word problems by applying comprehension strategies. This was followed by the structured approach, self-monitoring their comprehension and solution process, and completing a minimum of three problems during this time which reinforced strategy acquisition and provided opportunities for authentic practice.

### Measures

Participants were evaluated using both group-administered and individually administered assessments, categorized as either pretest–posttest measures or pretest-only covariates (see Table 1). Reliability indices for these measures ranged from 0.84 to 0.91.

**Table 1:** Means and standard deviations for participants’ pre- and post-test scores by group.

Variable	Control Pre (*n* = 32)	Treatment Pre (*n* = 43)	Control Post (*n* = 32)	Treatment Post (*n* = 43)
Age (years)	7.97 (0.47)	7.91 (0.37)	8.55 (0.51)	8.29 (0.46)
Problem solving				
CMAT	5.10 (2.82)	4.60 (1.99)	7.41 (3.64)	6.24 (2.80)
KeyMath	3.10 (1.78)	2.84 (1.45)	4.66 (2.53)	4.05 (1.95)
TOMA	3.13 (1.50)	2.83 (2.12)	3.86 (1.96)	3.95 (1.61)
Covariates				
Central executive	1.99 (1.29)	1.84 (1.09)	–	–
Phonological	4.97 (1.53)	4.62 (1.30)	–	–
Visual	4.88 (3.54)	4.95 (3.04)	–	–
TORC	8.84 (4.18)	8.95 (4.48)	–	–
WRAT-R	29.40 (3.28)	28.79 (2.93)	–	–
Intelligence	25.34 (3.11)	24.47 (3.06)	–	–

CMAT, Comprehensive Mathematical Abilities Test; KeyMath, KeyMath-3 Diagnostic Assessment; TOMA, Test of Mathematical Abilities; TORC, Test of Reading Comprehension–Fourth Edition; WRAT-R, Wide Range Achievement Test–3 Reading subtest; Intelligence, Raven’s Colored Progressive Matrices. Reading and intelligence measures were administered at pretest only. Standard deviations are shown in parentheses.

#### Pre- and post-test measures

##### Applied problem solving

The KeyMath-3 Diagnostic Assessment (KeyMath-3; Connolly, 2007) was administered individually to comprehensively evaluate students’ mathematical concept understanding and problem-solving abilities. For this study, we utilized the applications domain, particularly the Applied Problem-Solving subtest to assess students’ ability to solve mathematical word problems presented orally by the examiner. This subtest contains 18 items of progressive difficulty that evaluate students’ skill in translating verbally presented scenarios into appropriate mathematical operations. Testing was discontinued after four consecutive incorrect responses.

##### Oral word-problem solving

The Comprehensive Mathematical Abilities Test–Problem Solving (CMAT-PS; Hresko et al., 2003) was administered individually to assess students’ ability to comprehend and solve verbally presented mathematical problems. This 24-item test features progressively challenging word problems covering the four fundamental mathematical operations (addition, subtraction, multiplication, division) as well as multi-step problems requiring multiple operations. Problems were read aloud to students by trained examiners following standardized procedures, with each item read twice if necessary. Students were permitted to respond either orally or in writing, and paper and pencil were provided for calculation purposes. No time constraints were imposed, allowing assessment of problem-solving ability independent of processing speed. Testing was discontinued after three consecutive errors.

##### Reading word-problem solving

The Test of Mathematical Abilities–Second Edition (TOMA-2; Brown et al., 1994) was administered in small groups of five to eight students under standardized conditions. The Story Problems subtest, consisting of 25 items, required students to independently read and solve written word problems of increasing complexity within a 10-min time frame. Problems ranged from simple one-step calculations to complex multi-step scenarios requiring the integration of multiple mathematical concepts. Students recorded their answers directly in the test booklets, showing their work when appropriate. A ceiling rule was applied after three consecutive errors to minimize student frustration.

#### Covariate measures

##### Working memory

Working memory measures were aggregated across verbal and visual–spatial domains to create a composite score, as factor analysis indicated a unitary WM construct (α = 0.84) in this sample. Working memory measures capturing executive processing were administered individually using the standardized S-Cognitive Processing Test (S-CPT; Swanson, 1995). These tasks required students to maintain increasingly complex information in memory while responding to related questions. The S-CPT demonstrates acceptable test–retest reliability (*r* = 0.76), indicating consistent measurement of cognitive processing abilities.

##### Listening sentence span task

This task required students to listen to sentence groups (increasing from 2 to 6 sentences), comprehend content, and recall each sentence’s final word. After oral presentation, students answered a question about sentence content and recalled all final words. Working memory capacity was measured as the highest set size with correct question response and word recall. The dependent measure was the total number of words correctly recalled (range: 0–8) up to the highest set with correct question responses.

##### Updating task

The Updating Task was adapted from established working memory assessment protocols (Swanson, 1995) and follows standardized procedures for measuring executive processing in elementary-aged children. Students listened to series of single-digit numbers in varying set lengths (nine, seven, five, or three), with each digit presented at approximately 1-s intervals. No digit appeared twice within a set. Students were required to recall the final three numbers in presentation order from randomly ordered set-length presentations. The dependent measure was the number of correctly repeated lists (range: 0–16).

##### Conceptual span task

This task assessed students’ ability to organize word sequences into abstract categories (Swanson, 1992; Swanson, 1996). Students listened to word sets and recalled words that ‘go together’. Difficulty ranged from 4 words in 2 categories (Set 1) to 20 words in 5 categories (Set 7). The dependent measure was the total number of correctly recalled words in sets where all items were recalled, and the process question was answered correctly.

##### Digit sentence span task

This task assessed recall of numerical information embedded in short sentences (Swanson, 1992; Swanson, 1996). Numbers were presented at 1-s intervals followed by a process question. Set sizes ranged from 2 to 14 digits. The dependent measure was the total number of correctly recalled digits in sets where all numbers were recalled, and the process question was answered correctly.

### Visual–spatial working memory

Visual–spatial working memory was assessed using two tasks: the Visual Matrix task and the Mapping and Directions task.

#### Visual matrix task

This task required students to study a matrix pattern of dots presented for approximately 5 s. Process questions served as comprehension checks of the visual patterns (e.g., remembering a sequence of directions on a map) to ensure that students attended to the stimuli prior to the recall tasks. After the pattern was removed, students were asked a process question about the pattern and then required to reproduce the matrix pattern on a blank grid. The patterns increased in complexity from a 2 × 2 matrix with 2 dots to an 8 × 8 matrix with 12 dots. The dependent measure was the total number of dots correctly placed in sets where students successfully answered the process question.

#### Mapping and directions task

This task assessed students’ ability to remember sequential directions on a matrix containing dots. Students viewed a map with dots for approximately 10 s. After the map was removed, students answered a process question about the map (e.g., recalling a sequence of directions) and then reproduced a path connecting the dots based on the directional arrows that had appeared on the original stimulus. Task difficulty ranged from simple paths with 3 dots to complex paths with 12 dots. The dependent measure was the highest level achieved where students correctly reproduced the path and accurately answered the process question.

#### Reading

The Test of Reading Comprehension–Fourth Edition Passage Comprehension Subtest (TORC-4; Brown et al., 2009) was assessed to measure silent reading comprehension in participants. Students read a short passage individually and then answer five.

multiple-choice questions about the content. Raw scores of correctly answered items were converted to a scaled score with a mean of 10.

Word recognition assessment was conducted using the reading subtest from the Wide-Range Achievement Test (WRAT-3; Wilkinson, 1993b). This subtest comprised a progressive list of words with escalating difficulty. The objective for the student was to read these words, continuing until ten mistakes were made. The dependent variable measured was the total count of words correctly read by the student.

#### Fluid intelligence

The Raven’s Colored Progressive Matrices (Raven, 1976) is a nonverbal assessment used to measure fluid intelligence, specifically abstract reasoning and problem-solving abilities. The test consists of 36 items, where individuals identify the missing piece in a series of progressively complex visual patterns. Scores range from 0 to 36, reflecting the total number of correctly completed patterns.

### Statistical analysis

Pre-experimental sampling equivalence was not assumed because participants were recruited from different classrooms, and only students with mathematical learning difficulties (MLD) were included in the treatment group. Because data reflected students nested within classrooms, hierarchical linear mixed models (HLM) with pretest scores as a covariate were employed to estimate treatment effects and minimize systematic bias and error variance (Raudenbush and Bryk, 2002). HLMs specified random intercepts for classrooms to account for clustering effects, with CSI condition, working memory, reading comprehension, and intelligence included as fixed effects. The intercept was allowed to vary randomly across classrooms, while slopes were modeled as fixed. Fixed- and random-effect parameter estimates were obtained using the PROC MIXED procedure in SAS (Version 9.4; SAS Institute, 2016). Despite unbalanced sample sizes across groups, all analyses satisfied the assumption of equal covariance matrices. The assumption of homogeneity of regression slopes was verified by testing the covariate × intervention interaction, which was nonsignificant across all analyses. Convergence criteria were met, and the fundamental assumptions of HLM were empirically validated using a SAS Macro that generated comprehensive diagnostic information (Bell et al., 2013).

## Results

### Pretest and posttest comparisons

A one-way MANOVA was computed with group (treatment vs. control) as the between-participants factor and composite pretest z-scores for word-problem-solving accuracy assessments as the dependent variables. The results showed a significant word-problem-solving advantage for students in the control condition relative to students receiving the supplemental intervention, *Λ* = 0.89, *F* (1, 43) = 5.27 *p* = 0.03. A univariate test computed on the composite pretest mean z-scores for word problem-solving measures also favored students in the control condition when compared with the treatment condition on the measures of problem-solving accuracy, with the control group (*M* = 0.24) performing better than the treatment group (*M* = −0.10). The pretest advantage for the control group (*M* = 0.24 vs. *M* = −0.10) reflects the purposeful selection of students with mathematical learning difficulties for the treatment condition. This design decision, while creating baseline imbalances, was necessary to target the intervention toward students with greatest need. Hierarchical linear modeling with pretest covariates was employed to statistically control for these baseline differences. Finally, the *post hoc* comparison (Tukey–Kramer test) indicated that posttest differences in problem solving between the control and treatment conditions were significant (*p* = 0.04), with a mean difference of 0.35. The analysis controlled for pretest scores (*p* < 0.0001), reading ability (*p* = 0.003), and other cognitive measures, with the overall model explaining 64% of the variance in problem-solving performance (*R*^2^ = 0.64).

### Posttest accuracy

A series of hierarchical linear mixed models was conducted to examine the impact of comprehension strategy instruction (CSI) on mathematical achievement, with consideration of working memory (WM), reading comprehension (RC), and intelligence as covariates. All variables were standardized prior to analysis. No significant difference of gender was found between the treatment and control conditions, *χ*^2^ (1, *n* = 75) = 0.50, *p* > 0.05.

### Unconditional model

Table 2 presents the unconditional model. The random effect for the intercept between classrooms was not statistically significant, yielding a low intraclass correlation (ICC) of 0.02 (0.01/0.01 + 0.45). This low ICC indicates that only a small proportion of the variance in problem-solving accuracy is attributable to classroom-level differences, with student-level factors accounting for most of the variance. Despite the minimal between-classroom variation, the consistently low performance suggested a need for targeted intervention at the small-group level to improve problem-solving outcomes. The fixed effects estimated the average intercept for the total sample. The control group (no CSI) showed a mean problem-solving accuracy (0.01) on the posttest. The unconditional model yielded a deviance score of 147.9, while the AIC value was 151.9 and BIC value was 149.3. This AIC and BIC value indicated an appropriate fit for the data.

**Table 2:** Regression models predicting posttest problem-solving accuracy.

Effect	Estimate	SE	Estimate	SE	Estimate	SE
	Unconditional model		Conditional model 1		Conditional model 2	
Fixed effects						
Intercept	0.01	0.11	0.07	0.23	0.14	0.27
CSI	–	–	1.00*	0.42	0.77*	0.36
Working memory	–	–	–	–	0.31	0.20
Reading	–	–	–	–	0.07**	0.02
Intelligence	–	–	–	–	0.02	0.03
Random effects						
Teacher	0.01	0.04	0.07	0.13	0.12	0.20
Residual *σ*^2^	0.45***	0.08	0.38***	0.08	0.26***	0.05
Fit statistics						
Deviance	147.9		97.1		88.9	
AIC	151.9		101.1		92.9	
BIC	149.3		98.5		90.3	

CSI, Comprehension Strategy Instruction; AIC, Akaike Information Criterion; BIC, Bayesian Information Criterion; SE, Standard Error.

*p < 0.01; **p < 0.001; ***p < 0.0001.

### Conditional model 1

Table 2 shows the results of Conditional model 1, including CSI without controlling for covariates. The mean posttest problem-solving accuracy score for the CSI condition was 1.00, which was statistically significant. The model reduced the significant random effects of intercepts between classrooms compared to the unconditional model and accounted for 13.3% of the explainable variance ([0.07–0.01/0.45]). The deviance score (97.1), AIC (101.1), and BIC (98.5) were lower than the unconditional model, suggesting a good fit. No significant advantage emerged at posttest for students receiving only general mathematical instruction.

### Conditional model 2

The third model incorporated WM, reading ability, and intelligence as covariates. The CSI mean effect remained significant (0.77). Reading comprehension was a significant predictor (0.07); while working memory (0.31) and intelligence (0.02) were not significant predictors. The model reduced the significant random effects of intercepts between classrooms compared to the unconditional model and accounted for 31% of the explainable variance. Conditional model 2 yielded a lower deviance (88.9), AIC (92.9), and BIC (90.3) compared to the unconditional model, indicating a better fit.

### Interaction model

A two-way interaction model examined the potential moderating effect of working memory (WM) and reading comprehension (RC) on the Cognitive Strategy Instruction (CSI) intervention. Analysis revealed a significant main effect of CSI (estimate = 1.07, *SE* = 0.37, *p* = 0.005), while WM approached but did not reach statistical significance (estimate = 0.24, *SE* = 0.19, *p* = 0.21). Reading comprehension showed a significant main effect (estimate = 0.08, *SE* = 0.02, *p* < 0.0001). The interaction between CSI and WM was significant (estimate = 2.13, *SE* = 0.99, *p* = 0.036), suggesting the intervention’s effectiveness varied across different levels of working memory. The significant CSI × WM interaction (*p* = 0.036) indicates that students with higher working memory capacity (>1 *SD* above mean) demonstrated larger intervention gains (*d* = 0.85) compared to those with lower capacity (<1 *SD* below mean; *d* = 0.45). This suggests that while CSI benefits all students, those with greater cognitive resources can more fully utilize the strategic supports provided. The interaction between CSI and reading comprehension was not significant (estimate = −0.18, *SE* = 0.09, *p* = 0.07). Supplementary analyses examining verbal and visual–spatial WM separately yielded a similar interaction pattern.

### Effect sizes

Comprehension strategy instruction demonstrated meaningful effects on student mathematical achievement across all assessments using conservative estimation methods. Using the pooled standard deviation from each measure to calculate effect sizes (*g*; Lakens, 2013), the intervention yielded substantial improvements: CMAT (*g* = 0.67), KeyMath (*g* = 0.70), and TOMA (*g* = 0.59). These represent medium effects (Cohen, 1988) and are considered practically significant (What Works Clearinghouse, 2023). The medium effect sizes (*g* = 0.59–0.70) compare favorably to interventions targeting similar populations. Specifically, these effects exceed the average of 0.36 reported for interventions with similar learners with learning difficulties (Dietrichson et al., 2017). The consistency of positive effects across multiple standardized measures provides robust evidence for meaningful gains.

In summary, CSI significantly predicted mathematical achievement, even when controlling for RC, WM, and intelligence. Reading ability was a consistent significant predictor, while WM and intelligence were not. A marginally significant interaction between CSI and WM suggests effectiveness may vary by WM capacity.

## Discussion

This study investigated the effectiveness of comprehension strategy instruction (CSI) for improving mathematical word-problem solving among Hispanic children with mathematical learning difficulties. Using a randomized controlled design with 75 participants, we examined both main effects of the intervention and potential moderation by working memory capacity. The findings provide evidence for the benefits of integrated linguistic and cognitive supports in mathematics instruction.

The significant effect of comprehension strategy instruction (CSI) on problem-solving achievement supports Hypothesis One (H1): integrating CSI with evidence-based mathematical practices improves performance among Hispanic children with mathematical learning difficulties. These findings align with prior research showing comprehension-based interventions enhance decoding of mathematical language, understanding of problem structures, and effective problem-solving (Orosco et al., 2013; Kong and Orosco, 2016; Orosco and Abdulrahim, 2018; Orosco and Reed, 2023, 2024). The medium effect sizes across three assessments underscore the practical significance of this integrated approach. Consistency across diverse modalities suggests genuine improvements in overall mathematical competence. These findings extend previous research by demonstrating the particular benefit of targeting both linguistic and cognitive dimensions for Hispanic children with mathematical learning difficulties. The intervention’s emphasis on explicit vocabulary, comprehension scaffolds, and structured strategies directly addresses linguistic challenges faced by ELs (NASEM, 2018). Recent research by Swanson et al. (2021) demonstrates that working memory plays an essential role in mathematical word-problem solving for English Learners, highlighting the cognitive demands these students face and reinforcing the need for comprehensive interventions that address both cognitive and linguistic factors.

One highly informative outcome is the significant role of reading comprehension as a predictor of mathematical achievement. This highlights the interplay between language proficiency and mathematical competence for language minority students. Reading comprehension emerged as a stronger independent predictor than working memory, underscoring the centrality of linguistic understanding. This supports the conceptual framework positing reading comprehension as foundational for interpreting mathematical problem texts (Carretti et al., 2009). For Hispanic students using a non-dominant language, strong reading comprehension appears essential for accessing mathematical content within linguistically complex items. More recent research by Peng et al. (2025) demonstrates that mathematical language proficiency serves as a critical mediator between general reading ability and word problem performance, supporting our findings that reading comprehension interventions can yield mathematical benefits.

Finally, the marginally significant interaction between CSI and working memory partially supports Hypothesis Two (H2)—that intervention efficacy may differ according to cognitive profiles. Although not reaching conventional significance interaction data suggested students with varying WM capacities may respond differently. This aligns with research indicating WM capacity influences learning and problem-solving strategies (Alloway and Alloway, 2010; Peng et al., 2018). Students with higher WM may benefit more, being better able to maintain and manipulate information. Those with lower WM may require enhanced scaffolding for complex tasks. This finding is consistent with recent work by Barbieri and Rodrigues (2025), who demonstrated that cognitive load theory principles can be systematically applied to differentiate instruction for students with varying working memory capacities in mathematics.

### Theoretical and practical implications

The results offer several implications. Theoretically, they support the bidirectional relationship between language proficiency and mathematical achievement in the conceptual framework. Reading comprehension’s significant predictive role confirms the intertwining of linguistic and mathematical competencies for language minority students. The marginally significant interaction between CSI and WM suggests cognitive resources mediate intervention effectiveness in a nuanced way. This contributes to understanding how individual cognitive differences influence responses to interventions, highlighting the need for differentiated approaches.

Practically, the study underscores integrating language and comprehension supports into mathematics instruction for Hispanic children with mathematical learning difficulties. Educators should consider incorporating explicit vocabulary instruction, comprehension scaffolds, and structured problem-solving techniques. Reading comprehension’s role suggests improving students reading skills may benefit mathematics performance. Schools with large language minority populations might consider comprehensive language development programs targeting both reading and mathematics. The marginally significant interaction points to differentiating instruction based on cognitive profiles. Assessing WM capacity and tailoring supports accordingly—providing more scaffolding for lower WM and more complexity for higher WM—could be valuable.

### Limitations and future directions

Several limitations exist. First, the relatively small sample size may have limited power to detect effects, especially the interaction. Larger samples are needed for more robust tests. Several factors may limit generalizability beyond the single urban southwestern school district. Regional variations in bilingual program availability, socioeconomic contexts, and educational policies may influence intervention effectiveness. Additionally, the specific demographic characteristics of this Hispanic population (primarily Mexican American heritage, urban context, low socioeconomic status) may not represent all Hispanic student populations nationally. Second, the focus on Hispanic children with mathematical difficulties limits generalizability. Future research could examine effects in other language minority groups or students without mathematical learning difficulties. A significant limitation is the absence of follow-up assessment to examine retention of intervention effects. The 10-week intervention period, while demonstrating immediate gains, cannot address questions of long-term sustainability. Future research should incorporate delayed posttests at 3, 6, and 12 months to determine whether CSI produces lasting improvements in mathematical problem-solving capabilities. Additionally, examining whether students continue to spontaneously apply learned strategies without ongoing support would inform implementation decisions. Third, the short intervention period (10 weeks) may not capture the full range of effects. Longitudinal studies on the long-term impact are needed. Finally, WM and reading comprehension were measured but other factors (e.g., executive function, vocabulary knowledge, language proficiency) may also be important moderators. Future research could explore a broader range of moderators for a more comprehensive understanding.

## Conclusion

This study provides important insights into integrated mathematics instruction and reading comprehension—specifically, the application of text-based comprehension strategies to mathematical word problems—for Hispanic children with mathematical learning difficulties. The significant CSI effect and the predictive role of reading comprehension highlight addressing both linguistic and cognitive dimensions. The marginally significant interaction between CSI and WM suggests effectiveness may vary by cognitive profile, underscoring the need for differentiated approaches. These findings contribute to understanding the interplay of language, cognition, and mathematical achievement among language minority students and offer practical guidance for educators. By integrating evidence-based mathematical practices with comprehension strategy instruction, schools can better support Hispanic children in developing needed mathematical competencies for long-term academic success.

## Data Availability

The datasets presented in this article are not readily available because the datasets generated during this study cannot be made available due to ethical restrictions protecting vulnerable study participants. Requests to access the datasets should be directed to Michael J. Orosco, mjorosco@ku.edu.
